# Identification of a Novel Recycling Sequence in the C-tail of FPR2/ALX Receptor: ASSOCIATION WITH CELL PROTECTION FROM APOPTOSIS*

**DOI:** 10.1074/jbc.M114.612630

**Published:** 2014-10-17

**Authors:** Dawn Thompson, Simon McArthur, James N. Hislop, Roderick J. Flower, Mauro Perretti

**Affiliations:** From the ‡ William Harvey Research Institute, Barts and The London School of Medicine and Dentistry, Queen Mary University of London, Charterhouse Square, London EC1M 6BQ, United Kingdom and; the § School of Medical Sciences, College of Life Sciences and Medicine, University of Aberdeen, Institute of Medical Sciences, Foresterhill, Aberdeen AB25 2ZD, Scotland, United Kingdom

**Keywords:** Apoptosis, Arrestin, Cell Sorting, Endocytosis, G-protein-coupled Receptor (GPCR), Receptor Recycling

## Abstract

Formyl-peptide receptor type 2 (FPR2; also called ALX because it is the receptor for lipoxin A_4_) sustains a variety of biological responses relevant to the development and control of inflammation, yet the cellular regulation of this G-protein-coupled receptor remains unexplored. Here we report that, in response to peptide agonist activation, FPR2/ALX undergoes β-arrestin-mediated endocytosis followed by rapid recycling to the plasma membrane. We identify a transplantable recycling sequence that is both necessary and sufficient for efficient receptor recycling. Furthermore, removal of this C-terminal recycling sequence alters the endocytic fate of FPR2/ALX and evokes pro-apoptotic effects in response to agonist activation. This study demonstrates the importance of endocytic recycling in the anti-apoptotic properties of FPR2/ALX and identifies the molecular determinant required for modulation of this process fundamental for the control of inflammation.

## Teaser

**Background:** FPR2/ALX is important in the resolution of the immune response.

**Results:** Truncation of the C-tail results in attenuated recycling, receptor degradation, and enhanced apoptosis.

**Conclusion:** The FPR2/ALX contains a C-terminal motif mediating receptor recycling following endocytosis providing protection against cellular apoptosis.

**Significance:** This work identifies a novel transferable recycling sequence that controls GPCR trafficking and describes the cellular processes of the pro-resolution phase of the inflammatory response.

## Introduction

The inflammatory response is a defense mechanism developed to protect the host following an injury or infection. Once triggered, a series of soluble and cellular “pro-inflammatory” events is initiated resulting in swelling, redness, and pain. This initial phase is followed by a pro-resolving process, serving to restore tissue homeostasis (1). Failure of resolution leads to chronic inflammation and is implicated in pathologies including colitis (2) and asthma (3). One important group of receptors regulating these processes consists of the formyl peptide receptors (FPRs),^3^ which belong to the G-protein-coupled receptor (GPCR) superfamily. GPCR signaling modulates a variety of responses, from leukocyte activation and migration, to apoptosis and phagocytosis. At the intracellular level, GPCR signaling is controlled by a series of molecular events such as phosphorylation, arrestin interaction (which also initiates G-protein-independent signaling), desensitization, and endocytosis that serve to titrate signaling at the cellular level (4). There is limited understanding of the relationship between functional responses and their association with specific intracellular events, especially when considering receptor endocytosis and recycling.

The human FPR family consists of three intriguing members that bind a plethora of ligands to evoke several, often apparently opposite, effects (for recent review, see Ref. 5). The receptor that binds serum amyloid A, annexin-A1 (AnxA1), as well as the bioactive lipid mediators lipoxin A_4_ (LXA_4_) and resolvin D1 is termed FPR2/ALX (where the designation ALX refers to LXA_4_ receptor (6)). Both FPR1 and FPR2/ALX undergo agonist-induced endocytosis mediated via distinct mechanisms. Interestingly, FPR1 endocytosis is independent of either clathrin-mediated (7) or arrestin-mediated pathways (8–10), whereas the internalization of FPR2/ALX seems dependent on both (11, 12). Both receptors are rapidly recycled back to the plasma membrane following agonist removal; however, although FPR1 recycling requires the binding of both arrestins and adaptor protein 2 (AP2) (10, 13), little is known about the FPR2/ALX recycling process.

Numerous studies have implicated the C-tail of GPCRs in the regulation of cellular signaling and endocytosis (4), containing motifs that serve to bind sorting proteins and influence receptor fate. A series of elegant studies has systematically investigated specific clusters of serine and threonine residues within the C-tail of both the FPR1 (14–17) and the FPR2/ALX (18), demonstrating that, similar to other GPCRs, phosphorylation of specific serines in the region was critical for efficient phosphorylation and desensitization of either receptor. Whether these residues are important for regulating endocytosis or deciding cellular fate has yet to be elucidated.

Using the information previously reported for phosphorylation sites in FPR1 (14–17) and FPR2/ALX (18), we sought to investigate the importance of the C-tail in FPR2/ALX recycling following endocytosis in response to its potent agonist WKYMVm (W peptide). We identify a specific sequence in the C-terminal tail of FPR2/ALX necessary for the efficient recycling of this receptor that also modulates cell sensitivity to apoptosis.

## EXPERIMENTAL PROCEDURES

### 

#### 

##### Drugs and Reagents

FLAG M1 and FLAG M2 antibodies were purchased from Sigma. Alexa Fluor® 594 IGg2b, Alexa Fluor® 647 antibody labeling kit, LysoTracker Red DND-99, and Lipofectamine 2000 were from Life Technologies. WKYMVm peptide was purchased from Tocris, and [d-Ala-2, d-Leu-5]-enkephalin (DADLE) was purchased from Sigma. Propidium iodide and annexin V kit were purchased from Cell Signaling Technology. Total and phospho-ERK 1/2 and JNK antibodies were purchased from Cell Signaling Technology.

##### Cell Culture Constructs and Transfections

cDNAs of human FPR2/ALX and FPR1 (Missouri S&T cDNA Resource Center) were amplified by PCR (PfuUltra hotstart turbo, Agilent Technologies) and ligated into N-terminal signal sequence FLAG-tagged vectors (gift from Mark Von Zastrow, University of California, San Francisco (UCSF)). All truncations, point mutations, and amino acid additions were introduced by site-directed mutagenesis (PfuUltra, Agilent technologies). EGFP-β-Arr1, EGFP-β-Arr2, and EGFP-RAB5 were kind gifts from Prof. Mark von Zastrow (UCSF). EGFP-Rab11 was a kind gift from Dr. Rey Carabeo (University of Aberdeen).

##### Cell Culture and Immunocytochemistry

Constructs were either transiently or stably expressed in HEK293 cells and grown to 50% confluency on glass coverslips coated in 1% gelatin. Receptors where co-expressed with enhanced green fluorescence protein (EGFP) constructs where indicated. Cells were fed with anti-FLAG M1 antibody (1:1000, 30 min) to label mature cell surface receptors (as described previously (19)) and incubated with agonist for indicated time points, then fixed, blocked, and permeabilized and stained with fluorescently conjugated secondary antibody (1:1000). For LysoTracker experiments, cells were serum-starved and incubated with 100 nm LysoTracker Red DND-99 for 2 h prior to experimentation. For co-localization of receptor constructs with β-Arr2 at 30 min of W peptide treatment, at least 30 cells from three independent experiments were analyzed and quantified according to the proportion of pixels that were both red and green using Pearson's rank coefficient as part of the ImageJ plugin Coloc 2 (rsb.info.nih.gov/ij/).

##### Flow Cytometry

Anti-FLAG M1 antibody was conjugated to Alexa Fluor® 647 dye according to the manufacturer's instructions. HEK293 cells expressing N-terminally tagged constructs were subjected to a modified version of antibody feeding and flow cytometry (20, 21). Cells were incubated for 30 min with M1 Alexa Fluor® 647 (1:1,000), to label only surface receptors, and then stimulated for 30 min with agonist. Cells were washed with PBS/EDTA (2 mm) to remove residual antibody from remaining surface receptors and either resuspended in PBS (containing Ca^2+^ and Mg^2+^) to measure endocytosis (represented by an increase in fluorescence, indicative of endocytosed receptor protected from the strip wash) or returned to the incubator for 15, 30, 60, or 90 min in PBS containing EDTA to measure recycling (as determined by a loss of fluorescence due to receptors returning to the plasma membrane and being stripped of their label). After all treatments, cells were pelleted, washed, and resuspended in PBS (containing Ca^2+^ and Mg^2+^). Samples were analyzed using a FACSCalibur (BD Biosciences) with 10,000 events being analyzed in all cases.

##### Biotin Protection Degradation Assay

HEK293 cells stably expressing N-terminal FLAG-tagged FPR2/ALX, N333-stop, P342-stop, or T346-stop were grown to 100% confluency and labeled with 3 μg/ml disulfide-cleavable biotin (Pierce) for 30 min at 4 °C (19). Cells were then placed in 5 ml of medium stimulated for 30, 90, or 180 min. All plates (except the 100%) were then washed in PBS, stripped (50 mm glutathione, 0.3 mm NaCl, 75 mm NaOH, 1% FBS) at 4 °C for 30 min (to remove remaining cell surface-biotinylated receptors), quenched (PBS containing 1 mm iodoacetamide, 0.1% BSA), and then lysed in immunoprecipitation buffer (containing 0.1% Triton X-100, 150 mm NaCl, 25 mm KCl, 10 mm Tris-HCl (pH 7.4), and protease inhibitors (Roche Applied Science)). Lysates were immunoprecipitated (anti-FLAG M2) overnight and incubated for 2 h with recombinant protein G-Sepharose (Life Technologies) and deglycosylated. Samples were resolved by SDS-PAGE and visualized with streptavidin overlay (VECTASTAIN ABC immunoperoxidase reagent, Vector Laboratories).

##### Apoptosis Assay

HEK293 cells stably expressing N-terminal FLAG-tagged FPR2/ALX or N333-stop were grown to 50% confluency in 6-well plates and either untreated or treated with W peptide for 5 h. Etoposide was used as a positive control. Cells were lifted in 0.5 ml of PBS/EDTA pelleted at 1000 rpm and resuspended in binding buffer containing annexin V and propidium iodide according to the manufacturer's protocol. Cells were analyzed on a FACSCalibur with 10,000 events being analyzed in all cases.

##### Phospho-ERK and Phospho-JNK Signaling

HEK293 cells stably expressing N-terminal FLAG-tagged FPR2/ALX or N333-stop were grown to confluency in 6-well plates and serum-starved for 4 h prior to experimentation. For time course experiments, cells were untreated or stimulated with 500 nm W peptide for 5, 10, or 30 min. For resensitization experiments, cells were i) untreated, ii) stimulated for 5 min with the agonist, iii) incubated for 30 min agonist and then re-challenged with vehicle or compound for 5 min, or iv) treated for 30 min with agonist, washed in PBS, and returned to the incubator for 90 min to allow receptor prior to 5 min of challenge with either vehicle or drug. In all cases, samples were lysed in 150 μl of hot 4× SDS sample buffer containing DTT, separated using SDS-PAGE electrophoresis, and transferred to nitrocellulose. Blots were then assessed for phospho-ERK 1/2 or phospho-JNK and then stripped (stripping buffer, Pierce) and reprobed for total ERK1/2 or JNK (Cell Signaling). Signal was detected using a Fusion SL image capture system (PEQLAB).

##### Statistics

Data were analyzed using one-way ANOVA followed by Bonferroni's *t* tests between corresponding time points using GraphPad Prism 6.0 software.

## RESULTS

### 

#### 

##### Mutation of Serine and Threonine Clusters within the C-tail of FPR2/ALX Results in Altered Arrestin Recruitment and Subcellular Receptor Localization following Endocytosis

**FIGURE 1.**
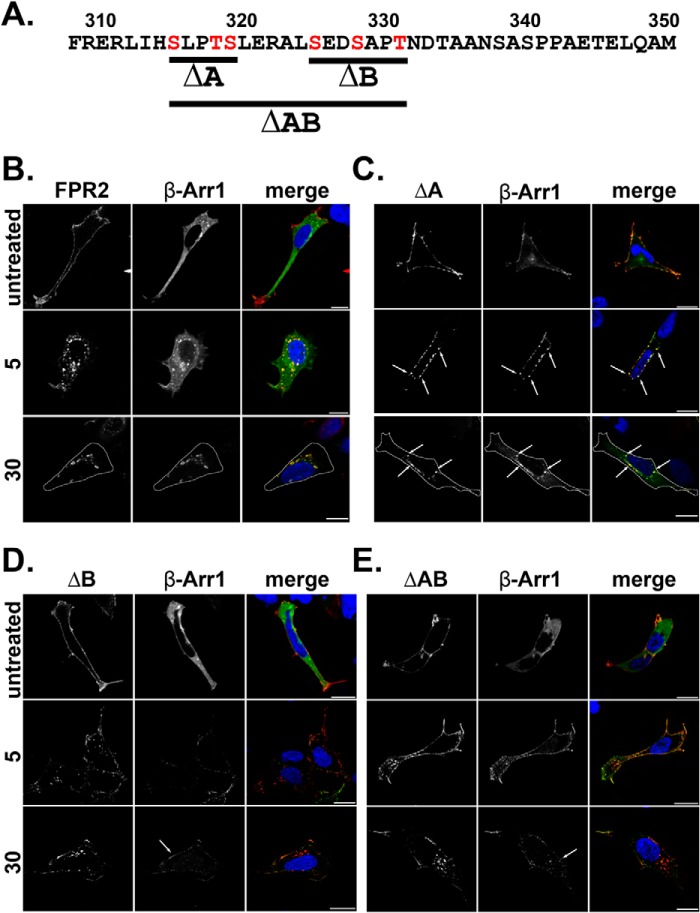
**Mutation of serines and threonines in the C-tail of FPR2 changes β-Arr1 binding patterns.** *A*, illustration of constructed phosphorylation mutants, ΔA, ΔB, and ΔAB, where *red* amino acids were mutated to alanines. *B–E*, HEK293 cells expressing N-terminally FLAG-tagged receptor constructs (FPR2, ΔA, ΔB, or ΔAB) and EGFP-tagged β-Arr1. Cells were fed with anti-FLAG M1 antibody to label receptor and treated as indicated (500 nm W peptide), then fixed, permeabilized, and incubated with secondary antibody prior to confocal microscopy. Representative images are shown with *scale bars* equal to 20 μm, and *dotted lines* mark the cell boundary.

Efficient receptor phosphorylation by GPCR-regulated kinases and arrestin recruitment have long been implicated as essential in the regulation of GPCR signaling, avoiding prolonged downstream activation (22). Subsequent internalization into endosomes and recycling to the plasma membrane serve as a means for receptor recovery, allowing further receptor activation and functional resensitization (4). We investigated the role of the C-tail in receptor endocytosis and recycling of the FPR2/ALX in response to W peptide (23), utilizing previous mutagenesis studies as a starting point. Specifically, we made use of the Ser/Thr clusters identified for human FPR2/ALX (18) and created three distinct mutants ΔA (Ser-316, Thr-319, and Ser-320), ΔB (Ser-326, Ser-329, and Thr-332), and ΔAB (Ser-316, Thr-319, Ser-320, Ser-326, Ser-329, and Thr-332), where serines and threonines were mutated to alanines (Fig. 1*A*, indicated in *red*).

**FIGURE 2.**
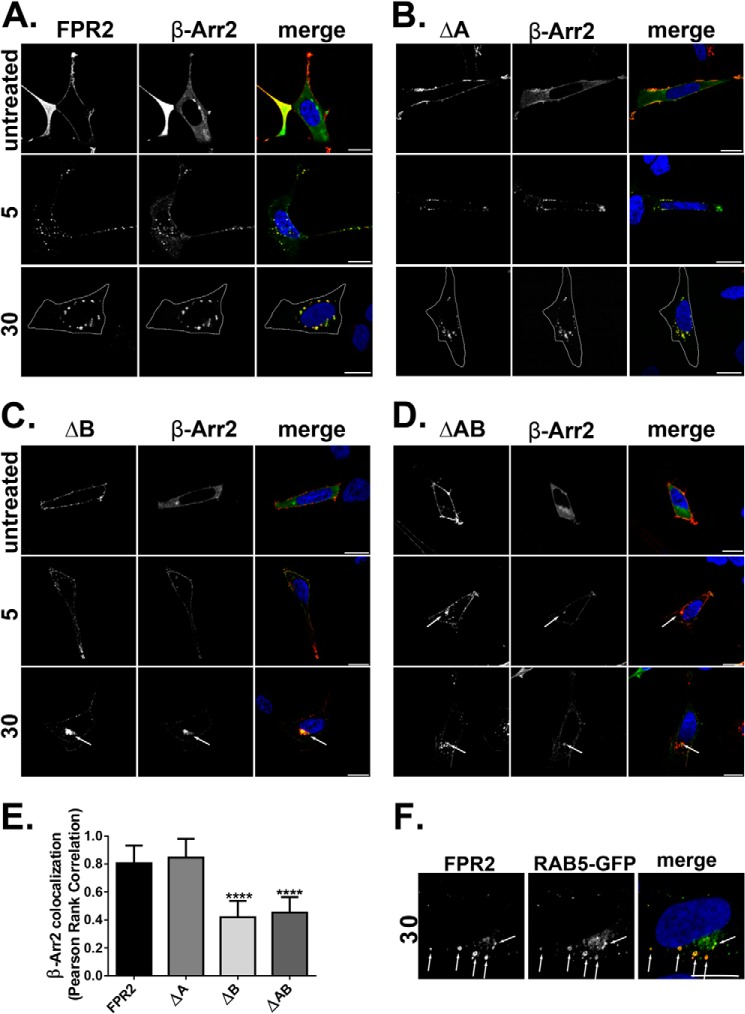
**Mutation of serines and threonines in the C-tail of FPR2 changes β-Arr2 binding patterns.** *A–D*, HEK293 cells expressing N-terminally FLAG-tagged receptor constructs (FPR2, ΔA, ΔB or ΔAB) and EGFP-tagged β-Arr2. Cells were fed with anti-FLAG M1 antibody to label receptor and treated as indicated (500 nm W peptide) and then fixed, permeabilized, and incubated with secondary antibody and visualized using confocal microscopy. *E*, quantification of β-Arr2 association with receptor constructs at 30 min of W peptide treatment expressed as Pearson's correlation coefficient. Data are represented as the mean co-localization of at least 30 cells performed on three separate occasions and analyzed using one-way ANOVA with Bonferroni's *t* test where ****, *p* ≤ 0.001 when compared with WT FPR2. E*rror bars* indicate mean ± S.D. *F*, cells co-expressing WT N-terminally FLAG-tagged FPR2 and EGFP Rab5 were fed with M1 antibody as in *A* and incubated for 20 min with W peptide. Representative images are shown with *scale bars* equal to 20 μm. Cell boundary is marked by a *dotted line*.

**FIGURE 3.**
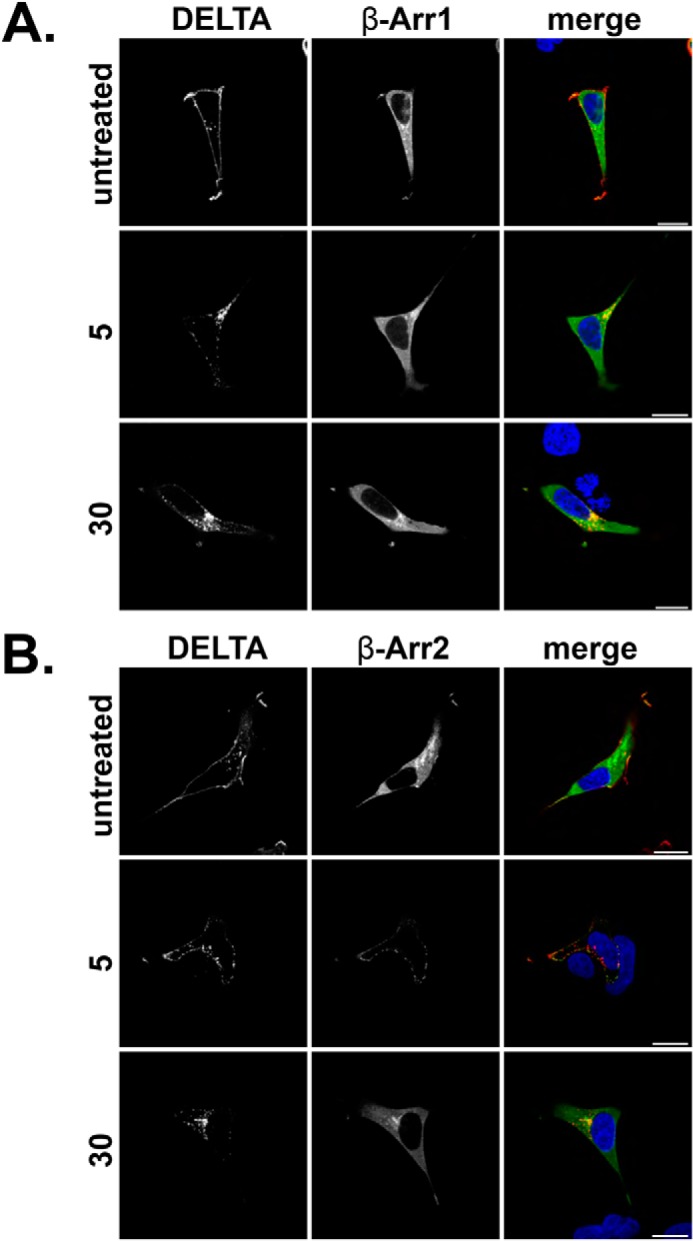
**DOR recruits β-Arr2.** *A* and *B*, HEK293 cells were transfected with N-terminally FLAG-tagged DOR and either EGFP β-Arr1 (*A*) or EGFP β-Arr2 (*B*) and fed with M1 antibody (1:1000) to label mature cell surface receptors. Cells were untreated or stimulated with DADLE (10 μm) for 5 or 30 min, fixed, permeabilized, and incubated with secondary antibody (anti-mouse IgG2b Alexa Fluor® 594, 1:1000) and visualized by confocal microscopy. Representative images are shown with *scale bars* equal to 20 μm. *DELTA*, δ-opioid receptor.

Typically, phosphorylation leads to arrestin recruitment followed by receptor endocytosis (22). To test whether these mutations influenced endocytosis and arrestin recruitment, HEK293 cells were transfected with WT FPR2/ALX, ΔA, ΔB, or ΔAB, co-expressed with either EGFP β-arrestin 1 (β-Arr1) or EGFP β-arrestin 2 (β-Arr2), and incubated with anti-FLAG M1 antibody to specifically label mature receptors on the plasma membrane (indicated in *red*) (19). In agreement with previous studies (11, 12), FPR2/ALX underwent rapid endocytosis recruiting both β-Arr1 and β-Arr2 within 5 min of W peptide agonist addition (optimal concentration of 500 nm) (Figs. 1*B* and 2A, *middle panels*), as indicated by a translocation from the plasma membrane to a punctate intracellular appearance. After 30 min, FPR2/ALX located in multiple enlarged endosomal structures dispersed throughout the cytoplasm and remained co-localized with both β-Arr1 and β-Arr2 (Figs. 1*B* and 2*A*, *lower panels*, indicated in *yellow*). On further investigation, the enlarged receptor/arrestin-containing structures we observed (Figs. 1*B* and 2*A*, *lower panels*) were Rab5-positive (Fig. 2*F*, *yellow staining*, see *arrows*), indicative of trafficking from the plasma membrane into the early sorting endosomes (24). In contrast, another rhodopsin-like GPCR, the δ-opioid receptor (DOR), showed robust recruitment of only β-Arr2 to the plasma membrane within 5 min of agonist exposure (10 μm DADLE), formed typically smaller puncta, and did not remain co-localized after 30 min as evident by the more diffuse cytoplasmic appearance (Fig. 3, *A* and *B*).

The recruitment pattern observed for FPR2/ALX was typical to that reported for the angiotensin (AT1R) and the vasopressin (V2R) receptors. Indeed, previous research has subdivided the rhodopsin-like GPCRs into two groups, the first exhibiting higher affinity for β-Arr2 and rapid dissociation of the receptor-arrestin complex (*i.e.* DOR), with the second showing equal affinities for β-Arr1 and β-Arr2 with prolonged, endosomal co-localization with arrestins following endocytosis (25). These data demonstrate that FPR2/ALX belongs to this second category.

**FIGURE 4.**
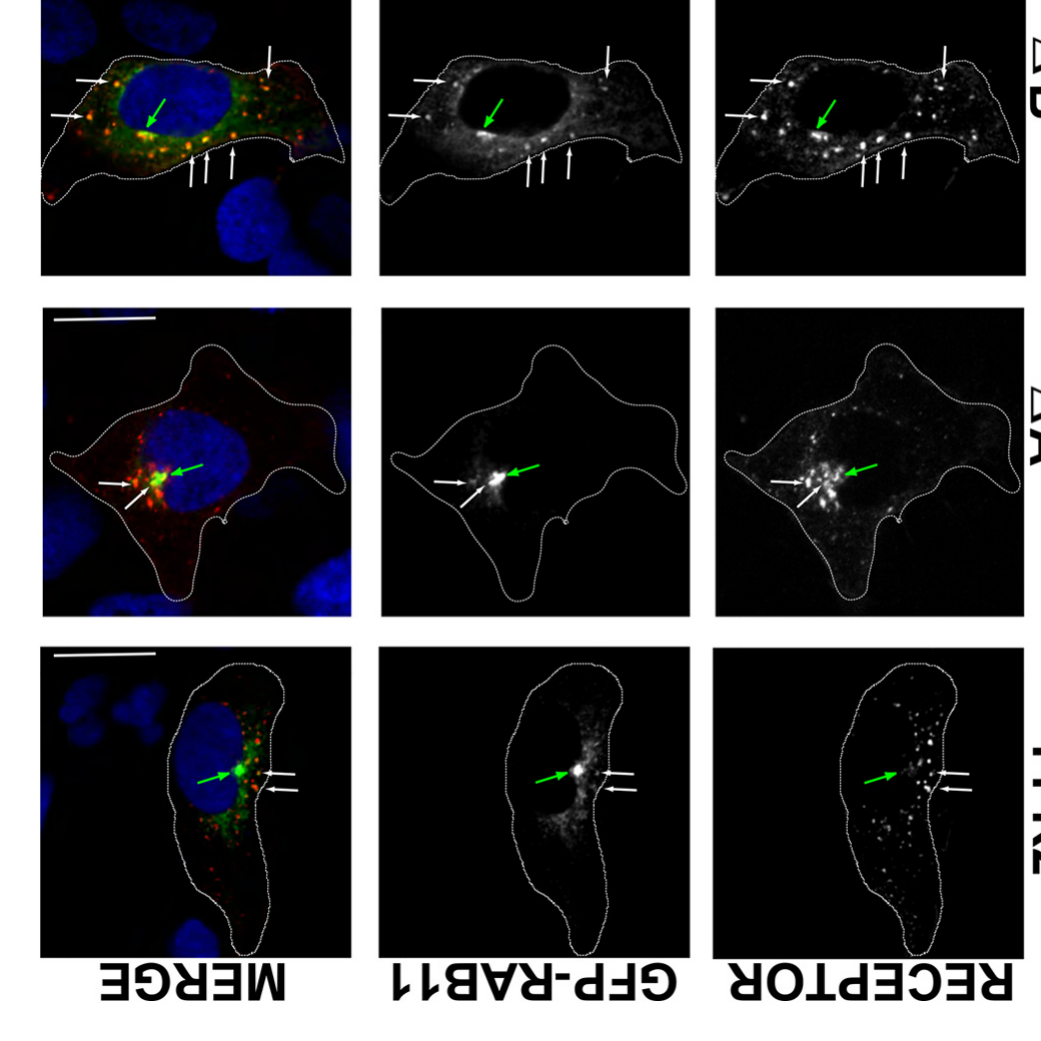
**Mutation of the ΔB residues confers retention in the Rab11 compartment.** Cells expressing WT N-terminally FLAG-tagged FPR2, ΔA, or ΔB were co-expressed with EGFP Rab11 and fed with M1 antibody as in Figs. 1 and 2 and incubated for 30 min with W peptide. Cells were then fixed, permeabilized, and incubated with secondary antibody (anti-mouse IgG2b Alexa Fluor ® 594, 1:1000) and visualized using confocal microscopy. Representative images are shown with *scale bars* equal to 20 μm (*green arrow* indicates the Rab11 recycling compartment, and *white arrows* show co-localization).

Comparable with WT FPR2/ALX, mutant ΔA underwent endocytosis and arrestin recruitment within 5 min, displaying similar enlarged endosomal structures after 30 min and co-localization with either β-Arr1 or β-Arr2 with equal affinity (Figs. 1*C* and 2*B*, *arrows*). However, mutant ΔB (Figs. 1*D* and 2*C*) or the combined mutant ΔAB (Figs. 1*E* and 2*D*) changed the appearance of both receptor and arrestin. Furthermore, there was significantly less β-Arr2 co-localization after 30 min of agonist treatment with mutant ΔB and ΔAB, whereas for ΔA, levels remained comparable with the FPR2/ALX control (Fig. 2*E*). Although these mutated FPR2/ALX forms underwent efficient endocytosis, they displayed a more clustered appearance typical of a perinuclear endocytic recycling compartment when expressing β-Arr2 (Fig. 2, *C* and *D*, *arrows*) (26). Furthermore, following translocation to the plasma membrane, both GFP β-Arr1 and β-Arr2 appear to remain at this location after 30 min with far less observed localized with receptors in these perinuclear compartments; this arrestin pattern is more typical of that observed with DOR (Fig. 3*B*) than with WT FPR2/ALX. Further investigation revealed that following 30 min of agonist treatment, the ΔB mutant was found to traffic to a compartment that showed distinctive co-localization with the Rab11 recycling endosome marker (Fig. 4, *arrows*). Although both the wild-type FPR2/ALX and the ΔA mutant show some co-localization with Rab11, this was much less pronounced than that seen for the ΔB (Fig. 4, *yellow staining*). Altogether, these data suggest that the Ser/Thr cluster in region B (Fig. 1*A*) is critical for continued arrestin interactions at the endosome, and mutation of these residues results in an altered post-endocytic location for the receptor targeting it to a Rab11-positive compartment before recycling back to the plasma membrane occurs.

##### Truncation of the C-tail of FPR2/ALX but Not the FPR1 Prevents Receptor Recycling

Arrestin has been implicated in the recycling of the homologous FPR1 (9) and other distinct GPCRs (27). Next, we used a flow protocol (20, 21) to determine whether the altered arrestin binding downstream of the FPR2/ALX phosphorylation mutants had any influence on receptor recycling following endocytosis and agonist removal. Mature cell surface receptors on the plasma membrane were incubated with M1-conjugated Alexa Fluor® 647 and either left untreated or stimulated with W peptide (500 nm; 30 min); a subsequent wash with PBS/EDTA removed any remaining surface staining to specifically label the endocytic pool (indicated by an increase in fluorescence when compared with the untreated strip control). Cells were then incubated for 15–90 min in the presence of PBS/EDTA to quantify receptor recycling; a loss of fluorescence indicates recycling and hence exposure to the PBS/EDTA strip at the cell surface.

**FIGURE 5.**
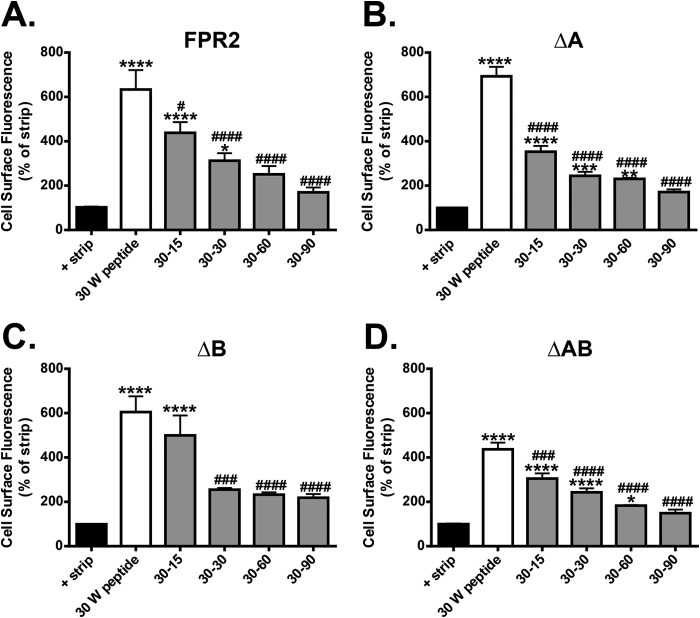
**Mutation of the serines and threonines in the C-tail of FPR2 has no effect on receptor recycling.** *A–D*, flow cytometry was used to analyze the endocytosis and recycling properties of FPR2, ΔA, ΔB, and ΔAB (see “Experimental Procedures”). HEK293 cells expressing either WT or mutant receptors were labeled with M1-conjugated Alexa Fluor® 647, stimulated with W peptide (500 nm) for 30 min, and either washed in PBS or returned to the incubator in the presence of PBS/EDTA for 15, 30, 60, or 90 min to monitor the degree of recycling. Data are represented as the mean of at least four independent experiments performed in duplicate analyzed using one-way ANOVA with Bonferroni's *t* test where ****, *p* ≤ 0.0001, ***, *p* ≤ 0.001, **, *p* ≤ 0.01, *, *p* ≤ 0.05 when compared with untreated controls or ####, *p* ≤ 0.0001, ###, *p* ≤ 0.001, #, *p* ≤ 0.05 when compared with W peptide-treated samples. E*rror bars* indicate mean ± S.D.

In agreement with a previous study (11), FPR2/ALX rapidly recycled back to the surface within 15 min with complete recycling at 60 min after wash, as shown by no significant difference from untreated samples (Fig. 5*A*). Interestingly, although both ΔA and ΔB mutants elicited internalization comparable with the WT, ΔAB exhibited a reduced fluorescence after 30 min, indicative of reduced internalization (despite expressing to comparable surface levels when compared with WT FPR2/ALX; Figs. 1*E* and 2*D*). This reduced internalization may explain the lack of Ca^2+^ desensitization previously observed by Rabiet *et al.* (18) in their investigation using similar phosphorylation-deficient mutations; furthermore, these data may suggest the requirement of other phosphorylation sites/regions within the receptor to mediate endocytosis.

Despite the observed differences in internalization, all mutant FPR2/ALX receptors were able to recycle the endocytosed pool as efficiently as the WT form, with complete recycling evident at 60 min after agonist removal. Indeed, the ΔA mutant appeared to recycle more efficiently than WT FPR2/ALX (Fig. 5*B*). Taken together with the data in Figs. 1 and 2, it appears that the Ser/Thr clusters do not determine the post-endocytic fate of the FPR2/ALX because efficient recycling is possible even in the absence of these residues. However, it is clear that the Ser/Thr clusters do play functional roles in endocytic trafficking, with phosphorylation potentially determining which compartment the receptor enters and how efficiently it recycles, rather than the absolute receptor fate *per se*.

**FIGURE 6.**
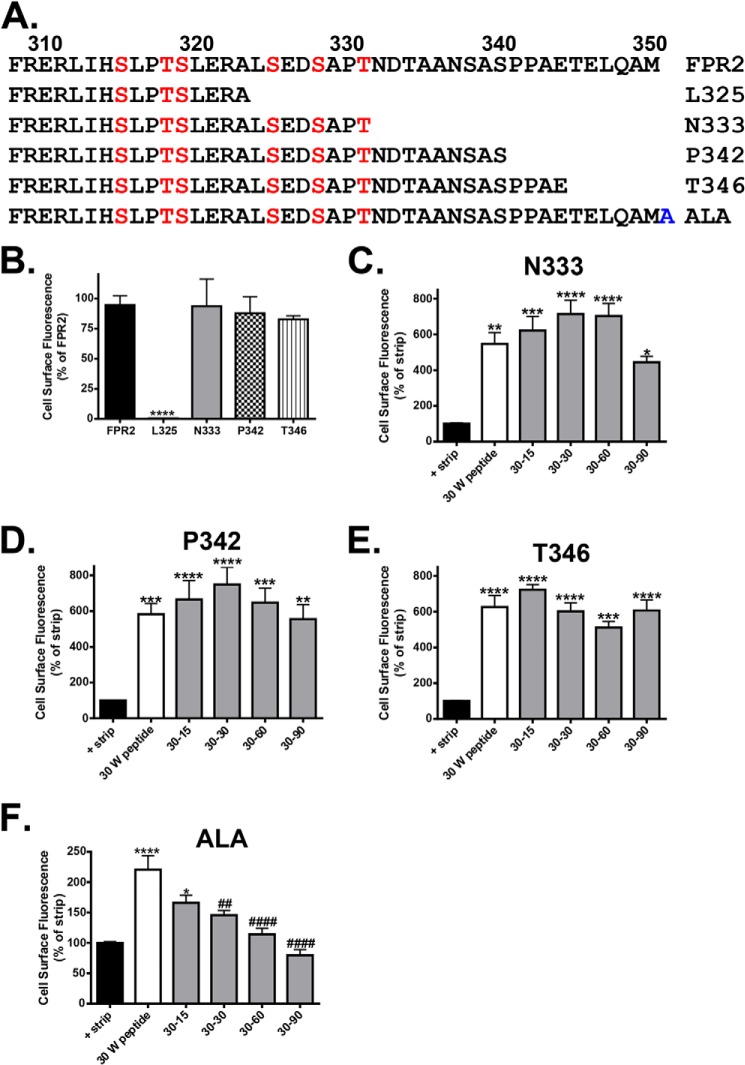
**Truncation of the FPR2 C-tail prevents receptor recycling.** *A*, illustration of truncation mutants where stop codons were introduced at amino acids Leu-325, Asn-333, Pro-342, or Thr-346 or the addition of a single alanine to the end of the C-tail (*ALA*). *B*, WT FPR2 or the truncation mutants were assessed for cell surface expression by flow cytometry. *C–F*, the endocytosis and recycling properties of N333-stop (*N333*), P342-stop (*P342*), T346-stop (*T346*), and ALA was analyzed by flow cytometry as in Fig. 5. Data are represented as the mean of at least four independent experiments performed in duplicate analyzed using one-way ANOVA with Bonferroni's *t* test where ****, *p* ≤ 0.0001, ***, *p* ≤ 0.001, **, *p* ≤ 0.01, *, *p* ≤ 0.05 when compared with untreated controls or ##, *p* ≤ 0.01, ####, *p* ≤ 0.0001 when compared with W peptide-treated samples. There were no significant differences in samples when compared with W peptide-treated samples. E*rror bars* indicate mean ± S.D.

GPCR recycling can be regulated by sequences present in the distal C terminus, with motifs identified for the μ-opioid receptor (28), β_2_-adrenergic receptor (β_2_AR) (29), and dopamine D1 receptor (D1R) (30) among others (4) where either the truncation of or the addition of epitope tags or amino acids to the C terminus resulted in attenuated receptor recycling. In an attempt to identify potential recycling motifs within the C-terminal region of FPR2/ALX, we made several constructs containing stop codons designed to prematurely truncate the C-tail at different residues (Fig. 6*A*) and used the modified method of flow cytometry (described for Fig. 5) to investigate their post-endocytic properties. It was observed that truncation at Leu-325 resulted in reduced receptor expression at the plasma membrane presumably due to structural changes in stability (Fig. 6*B*). Importantly, truncation of FPR2/ALX at N333-stop, P342-stop, or T346-stop showed no differences in receptor endocytosis, but receptor recycling was inhibited in all clones, as indicated by the maintenance of fluorescence (Fig. 6, *C–E*), rather than the decrease observed for the WT receptor (Fig. 5*A*). Also, the addition of an alanine to the end of the C terminus did not prevent recycling, distinguishing this new sequence from that seen in the β_2_AR (Fig. 6*F*).

**FIGURE 7.**
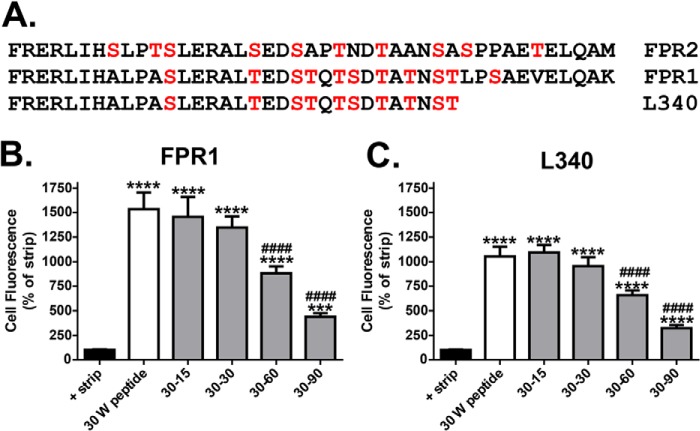
**Truncation of the FPR1 C-tail does not prevent receptor recycling.** *A*, alignment of FPR1 and FPR2 C-tail sequences and illustration of truncation mutant where a stop codon was introduced at amino acid Leu-340 in the FPR1 backbone. *L340*, L340-stop. *B* and *C*, the endocytosis and recycling properties of WT FPR1 and Leu-340 were analyzed by flow cytometry as in Fig. 5. Data are represented as the mean of at least four independent experiments performed in duplicate analyzed using one-way ANOVA with Bonferroni's *t* test where ****, *p* ≤ 0.0001, ***, *p* ≤ 0.001 when compared with untreated controls or ####, *p* ≤ 0.0001 when compared with W peptide-treated samples. E*rror bars* indicate mean ± S.D.

Next, we investigated whether a similar sequence exists within the FPR1 distal C terminus that differs from the FPR2/ALX by a single lysine (rather than a methionine, Fig. 7*A*). Consistent with previously published data (9), we found that FPR1 underwent extensive recycling following agonist washout (Fig. 7*B*) in a similar fashion to FPR2/ALX (Fig. 5*A*). Interestingly, in contrast to the FPR2/ALX, truncation of the C-tail in the FPR1 homologue did not attenuate receptor recycling (Fig. 7*C*) and instead exhibited a time-dependent decrease in fluorescence comparable with the WT FPR1 (Fig. 7*B*).

##### Recycling-deficient FPR2/ALX Results in Cellular Apoptosis through a Phospho-JNK-mediated Pathway

**FIGURE 8.**
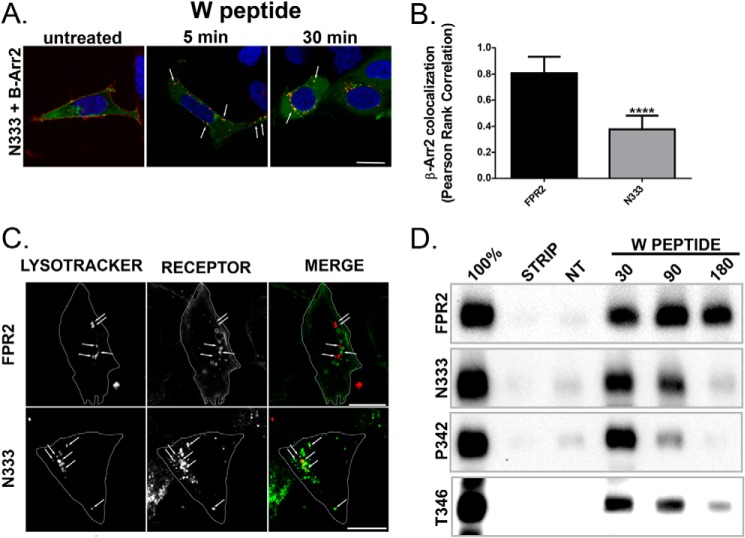
**Attenuated recycling leads to lysosomal targeting and receptor down-regulation.** *A*, HEK293 cells expressing N-terminally FLAG-tagged N333-stop and EGFP-tagged β-Arr2 were fed with anti-FLAG M1 antibody as in Fig. 2, treated with W peptide, and then fixed, permeabilized, and incubated with secondary antibody and visualized using confocal microscopy. Representative images are shown with *scale bars* equal to 20 μm, and *arrows* indicate examples of co-localization. *B*, quantification of β-Arr2 association with FPR2 and N333-stop (*N333*) at 30-min W peptide treatment expressed as Pearson's correlation. Data are represented as the mean co-localization of at least 30 cells performed on three separate occasions and analyzed using one-way ANOVA with Bonferroni's *t* test where ****, *p* ≤ 0.001 when compared with WT FPR2. E*rror bars* indicate mean ± S.D. *C*, HEK293 cells stably expressing FPR2 or N333-stop were labeled with anti-FLAG M1 antibody and LysoTracker and incubated for 90 min with 500 nm W peptide. Representative confocal images are shown with *scale bars* equal to 20 μm, and *arrows* indicate the lysosomal compartment. *D*, HEK293 cells stably expressing FPR2, N333-stop (*N333*), P342-stop (*P343*), or T346-stop (*T346*) were surface-biotinylated and either untreated or stimulated with 500 nm W peptide for 30, 90, or 180 min. Receptor fate was assessed after immunoprecipitation with anti-FLAG M2 antibody, subsequent separation by SDS-PAGE electrophoresis, and streptavidin overlay. The *100% lane* shows total surface receptor labeling, and the *STRIP lane* indicates the efficiency of the biotin cleavage. *NT*, non-treatment. Representative immunoblots are shown.

Given the changes in arrestin recruitment following mutation of phosphorylation sites in the C-tail of FPR2/ALX (Fig. 2), we assessed the ability of mutant N333-stop to recruit β-Arr2. Although showing some endosomal co-localization with β-Arr2 (Fig. 8*A*, *arrows*), these endosomes were smaller in size than that typically seen with the WT FPR2/ALX, and N333-stop exhibited significantly less β-Arr2 co-localization following 30 min of agonist treatment (Fig. 8, *A* and *B*), similar to that observed for the ΔB and ΔAB mutants (Fig. 2), perhaps indicating transition through the endosomal compartment.

Lack of receptor recycling following endocytosis could manifest as either an accumulation of receptor in endosomal compartments, such as in the truncated D1R (19, 30), or a change of fate whereby the receptor is transported for lysosomal degradation, as in the case of the truncated μ-opioid receptor (19, 28). HEK293 cells stably expressing either WT FPR2/ALX or mutant N333-stop were incubated with 500 nm W peptide and visualized for their co-localization with lysosomes (via LysoTracker red staining, Fig. 8*C*, *arrows*). After 90 min, N333-stop could be found co-localized with lysosomal compartments (Fig. 8*C*, *yellow staining*), unlike WT FPR2/ALX, which existed in distinct structures, and could also be observed having returned to the plasma membrane. Hence, N333-stop was trafficked away from the recycling compartments and targeted to the acidic lysosomes.

To investigate post-endocytic receptor stability following prolonged agonist treatment, we used HEK293 cells stably expressing FPR2/ALX or its corresponding mutants and made use of the biotin protection assay (see “Experimental Procedures”). By using this method, we could follow the fate of only the mature receptors on the plasma membrane, whereas endocytosed receptors are protected from the strip wash. WT FPR2/ALX remained stable after 180-min exposure to W peptide (500 nm), whereas all three mutant (N333-stop, P342-stop, and T346-stop) receptors showed reduced expression levels at the same time point, indicative of receptor degradation (Fig. 8*D*). Hence, truncation of FPR2/ALX not only prevented recycling following endocytosis in response to W peptide but also resulted in a change of fate whereby FPR2/ALX mutants were targeted to acidic lysosomal compartments and down-regulated following sustained ligand treatment.

**FIGURE 9.**
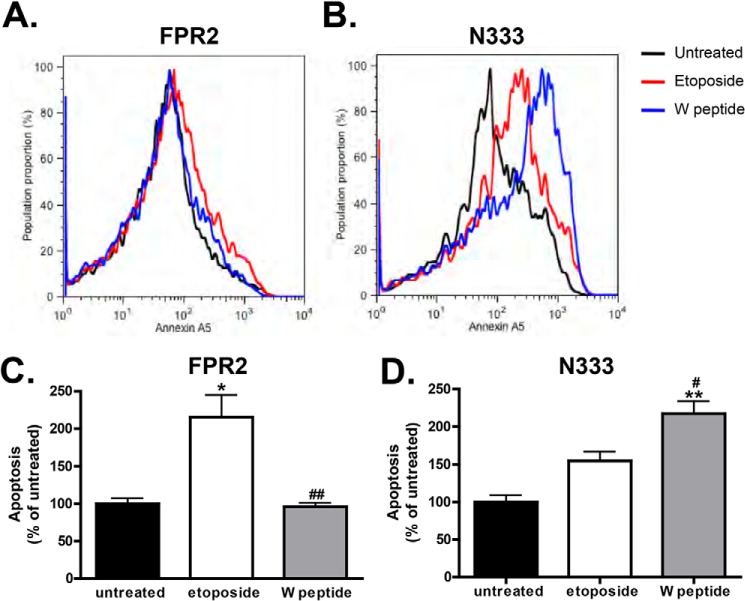
**Attenuated recycling leads to cellular apoptosis.** *A* and *B*, cells expressing either FPR2 (*A*) or N333-stop (*N333*) (*B*) were untreated or treated for 5 h with either etoposide or W peptide, stained with propidium iodide and annexin V, and analyzed using flow cytometry. A representative trace is shown. *C* and *D*, quantification of three experiments performed in duplicate and analyzed using one-way ANOVA with Bonferroni's t-tests. **, *p* ≤ 0.01, *, *p* ≤ 0.05 when compared with untreated controls. ##, *p* ≤ 0.01 or #, *p* ≤ 0.05 when compared with W peptide-treated samples. E*rror bars* indicate mean ± S.D.

Previous studies involving FPR1, using mouse embryonic fibroblast cells devoid of both β-Arr1 and β-Arr2, reported failure of this receptor to recycle with consequent cellular accumulation and apoptosis (10). We hypothesized that failure of FPR2/ALX to efficiently recycle in response to W peptide would also impact on cellular apoptosis. We used the classical annexin A5:propidium iodide flow cytometry protocols to measure the degree of apoptosis in FPR2/ALX WT and N333-stop mutant cells. Treatment of native FPR2/ALX for 5 h with W peptide resulted in minimal cellular apoptosis (Fig. 9, *A* and *C*), whereas apoptosis was markedly increased in cells overexpressing N333-stop, the recycling-deficient mutant (Fig. 9, *B* and *D*). Furthermore, N333-stop exhibited a higher basal apoptosis in untreated samples presumably due to accumulation following constitutive internalization and defective recycling. Hence, these data indicate that efficient internalization and recycling of FPR2/ALX is both required and necessary for protection against programmed cell death.

**FIGURE 10.**
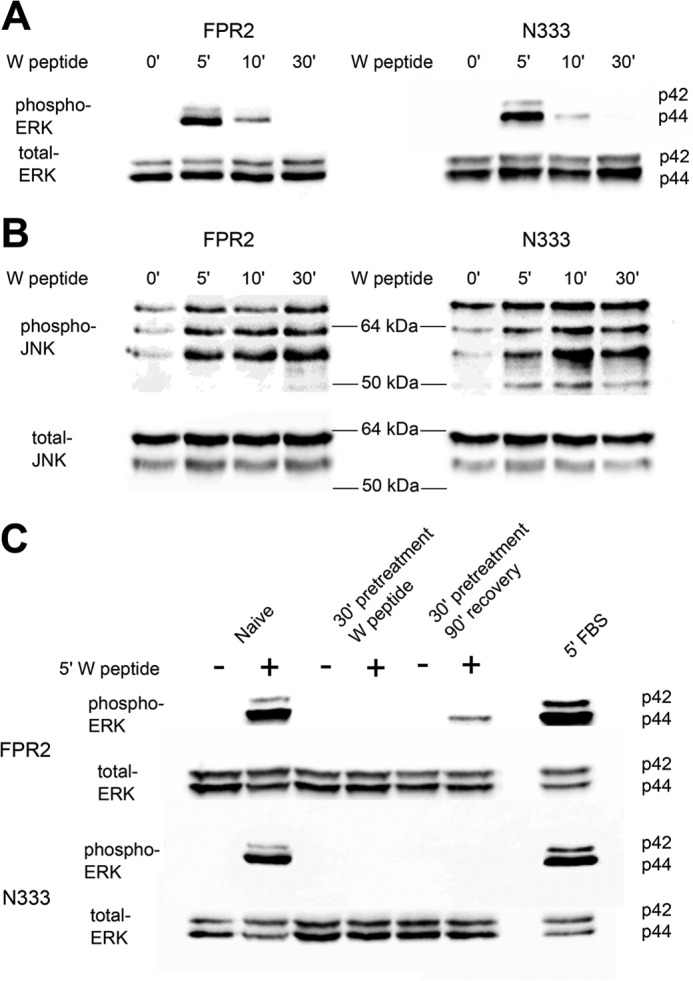
**MAPK signaling of FPR2 and N333-stop.** *A* and *B*, HEK293 cells stably expressing either N-terminally FLAG-tagged WT FPR2 or N333-stop (*N333*) were serum-starved 4 h prior to experimentation. Cells were untreated or stimulated with 500 nm W peptide for 5, 10, and 30 min, lysed, separated by SDS-PAGE, and probed for phospho-ERK 1/2 (*A*) or JNK (*B*), and then stripped and reprobed for total ERK 1/2 and JNK. *C*, for resensitization, phospho-ERK 1/2 was investigated where cells were either untreated, stimulated for 5 min, pretreated with agonist for 30 min, and then re-challenged with vehicle or drug (desensitized) or pretreated for 30 min, washed, and allowed to recover for 90 min at 37 °C before a final re-challenge for 5 min with agonist or vehicle (resensitization and recycling). Representative blots are shown of at least three independent experiments.

To elucidate the pathways responsible for increased apoptosis in N333-stop-expressing cells, phospho-ERK1/2 and phospho-JNK were investigated as possible signaling candidates (31). Indeed, previous work from this laboratory identified JNK phosphorylation to precede apoptosis in neutrophils through activation of an FPR1/FPR2/ALX heterodimer (31). Firstly, FPR2/ALX- or N333-stop-expressing cells were untreated or stimulated with W peptide for 5, 10, or 30 min. Both WT and mutant-expressing cells phosphorylated ERK 1/2 in a similar fashion, peaking at 5 min and undetectable by 30 min, indicative of receptor desensitization (Fig. 10*A*). In contrast to ERK 1/2 signaling, both FPR2/ALX and N333-stop showed increased and sustained activation of phospho-JNK up to 30 min (Fig. 10*B*).

Several studies have reported receptor recycling to the plasma membrane to be important in the recovery of the cellular response (32–35). To assess whether receptor recycling resulted in renewed signaling, phospho-ERK 1/2 was investigated in FPR2/ALX- and N333-stop-expressing cell lines. As observed previously, both receptors evoked robust phospho-ERK1/2 signaling after 5-min stimulation with W peptide (Fig. 10*C*). However, no signal could be detected after pretreatment for 30 min with agonist followed by a 5-min re-challenge, which is consistent with desensitization of the receptor followed by endocytosis and removal from the plasma membrane. Furthermore, following agonist washout and a 90-min recovery period, re-challenge of FPR2/ALX with agonist exhibited recovery of the phospho-ERK1/2 response, which was absent from N333-stop-expressing cells. These data are consistent with FPR2/ALX internalizing and recycling following agonist washout (Fig. 5*A*), allowing receptor resensitization and the ability to once again respond to ligand at the cell surface. In contrast, the lack of signal recovery observed in the N333-stop-expressing cell lines is consistent with the lack of recycling reported in Fig. 6*C*.

##### The FPR2/ALX C-tail Contains a Transplantable Recycling Motif

**FIGURE 11.**
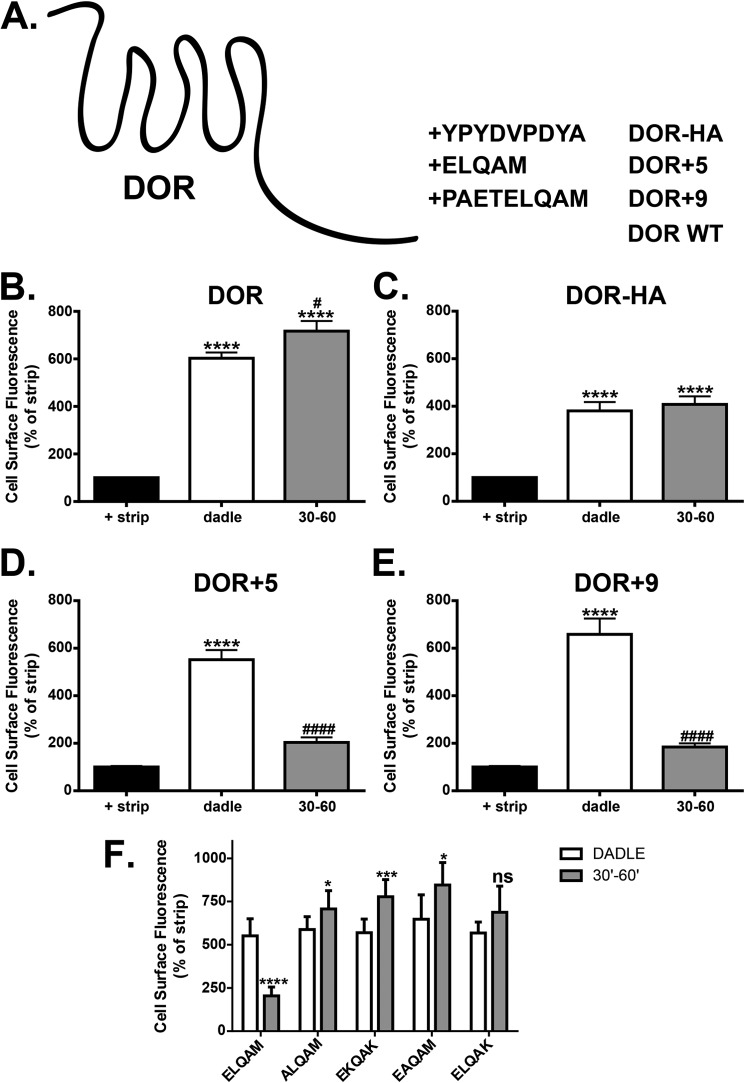
**FPR2 contains a specific motif that is able to facilitate recycling of the degrading δ-opioid receptor.** *A*, chimeras of DOR as introduced by PCR: DOR-HA (YPYDVPDYA), DOR+5 (ELQAM), and DOR+9 (PAETELQAM). *B–E*, cells transiently expressing WT or chimeric constructs were assessed for endocytosis and recycling by flow cytometry (see “Experimental Procedures”). Cells were labeled as in Fig. 5 and either untreated or stimulated for 30 min with DADLE (10 μm) and then washed with PBS/EDTA to assess acute endocytosis, or stimulated and returned to the incubator for 60 min in the presence of PBS/EDTA to assess receptor recycling. *F*, point mutations were introduced to the DOR+5 backbone to elucidate specific amino acids within this motif critical for receptor recycling. Data are represented as the mean of at three independent experiments performed in duplicate analyzed using one-way ANOVA with Bonferroni's *t* test where ****, *p* ≤ 0.0001, ***, **, *p* ≤ 0.01, *, *p* ≤ 0.05 when compared with untreated controls or ####, *p* ≤ 0.0001 or #, *p* ≤ 0.05 when compared with DADLE-treated samples. E*rror bars* indicate mean ± S.D.

Previous publications have reported that DOR undergoes down-regulation following chronic agonist exposure, with little evidence for recycling following agonist removal. Transplantation of a recycling motif from either the β_2_AR or the μ-opioid receptor was enough to reroute the endocytic trafficking of DOR and facilitate recycling of this receptor (28, 36). We sought to identify whether residues in the C terminus of the FPR2/ALX could also be transplanted onto DOR to evoke efficient recycling. Three constructs were made introducing several amino acids using PCR. These were the addition of the human influenza HA tag (YPYDVPDYA, DOR-HA) or the last five (ELQAM, DOR+5) or nine (PAETELQAM, DOR+9) amino acids from the distal portion of the FPR2/ALX C-tail (Fig. 11*A*). The addition of the HA tag to the C-tail of DOR (37), although eliciting a slight reduction in endocytosis, did not alter the recycling properties with the receptor failing to return to the plasma membrane, indicating that purely adding any tag to DOR was insufficient to elicit recycling (Fig. 11, *B* and *C*). In comparison, transplantation of either five or nine amino acids from the most distal portion of the C terminus of FPR2/ALX afforded receptor recycling following endocytosis (Fig. 11, *D* and *E*).

To further investigate this recycling motif, four further mutations were made within the DOR+5 backbone. Firstly, the methionine was mutated to a lysine to resemble the most distal portion of the FPR1 C-tail (*i.e.* ELQAK). Secondly, three further point mutations were made to the most conserved residues across the many FPR2 sequences in GenBank^TM^ (ALQAM, EKQAK, EAQAM). All mutations subsequently attenuated DOR recycling when compared with DOR+5 (Fig. 11*F*), indicating that these conserved amino acids are all required for maintaining a functional recycling sequence. There results demonstrate that the sequence identified in FPR2/ALX is a *bona fide* recycling sequence, being both necessary and sufficient for recycling, and is distinct from that of FPR1.

## DISCUSSION

FPR2/ALX has long been identified as an important mediator of the inflammatory response responding to activation by both pro-inflammatory and anti-inflammatory ligands. In particular, activation of this receptor is pivotal in the initiation of the anti-inflammatory, pro-resolution portion of this cascade. Failure of resolution prevents the return to cellular homeostasis, resulting in persistent inflammatory conditions. Therefore, understanding the molecular machinery of FPR2/ALX function is critical to understanding physiological and pathophysiological inflammatory processes.

We report new evidence that specific Ser/Thr clusters in the C-tail of FPR2/ALX are responsible for arrestin binding and targeting to specific subcellular cytoplasmic compartments. The data presented herein indicate that efficient interaction with arrestins ensures residence within the early, Rab5-positive endosome, as well as subsequent rapid recycling (mutant ΔA), whereas the absence of arrestin interaction (mutant ΔB) promotes transit through to a different, slower recycling compartment likely regulated by Rab11. It will be important in future studies to determine whether and how these recycling routes differ and what role the two routes play in the trafficking of the WT FPR2/ALX. Importantly, FPR2/ALX is able to efficiently recycle from both compartments, suggesting that the stable arrestin interaction observed does not determine the endocytic fate of the receptor. This is in stark contrast to that observed for FPR1 where mouse embryonic fibroblast cells devoid of both β-Arr1 and β-Arr2, although showing no inhibition of endocytosis, were unable to efficiently recycle to the plasma membrane (10) and instead remained within the Rab11 compartment. Of note, the FPR2/ALX ΔB mutant appears to be localized to a similar, Rab11, compartment following endocytosis but, unlike FPR1, is still able to recycle. Whether arrestin plays any subtle role at this stage remains to be determined.

We have identified for the first time a specific recycling motif at the distal portion of the C-terminal tail that, if removed, fails to influence endocytosis but instead attenuates the ability of this receptor to recycle, targeting it for degradation. Analysis of the sequences of several FPR2/ALX within GenBank indicates a consensus sequence of (D/E)φ*XX*φ (where φ is any hydrophobic residue), although this needs to be validated for other receptors. Alignment of FPR1 revealed that this receptor is missing the methionine at its most distal residue and instead possesses a lysine at this position. However, this lysine is neither necessary nor sufficient for recycling because truncation failed to attenuate return of the FPR1 to the plasma membrane. Indeed, mutation of any of these conserved residues within the distal C terminus of FPR2/ALX removes the ability of this sequence to transfer recycling onto the DOR. Interestingly, this sequence does not appear to be conserved among any other GPCRs, and thus identification of the recycling protein is an interesting area of study as it may represent a new target for therapeutic intervention.

The most widely understood GPCR recycling sequence is the type I PDZ ligand found on β_2_AR, which allows interaction with sorting nexin 27 and recycling mediated via the retromer (38, 39). The ELQAM sequence present on the FPR2/ALX, however, is not a canonical type I PDZ ligand, and the addition of an alanine residue did not inhibit recycling (in contrast to the β_2_AR (29)) altogether, suggesting the involvement of a different recycling pathway. It therefore remains to be seen whether any of the processes described for other receptors (e.g.SNX27, retromer, etc.) are involved in mediating FPR2/ALX recycling, although the fact that the conserved sequence cannot be identified in any other GPCRs might suggest the existence of novel interacting partner(s) that regulate the recycling of the FPR2/ALX localization.

It has long been established that GPCR endocytosis and recycling serve to modulate receptor signaling, thereby preventing overstimulation. More recent research has revealed that signaling via GPCRs may also be controlled via biased agonism of the receptor. Indeed, transient ERK 1/2 phosphorylation has been found to be G-protein-dependent, whereas more sustained signaling occurs via a β-Arr2-mediated pathway (40). In this study, we provide evidence for both ERK 1/2 and JNK activation, which have been previously reported to be G_i_-dependent, rather than an arrestin-mediated response (41). Additionally, Wagener *et al.* (10) report that arrestin-mediated recycling of FPR1 is paramount in the suppression of the apoptotic signal. Therefore, it seems that the important anti-apoptotic effect relies on the ability of the receptor to recycle and resensitize the receptor rather than ligand bias *per se*.

The cytoplasmic mechanism regulating recycling of FPR2/ALX therefore remains an important avenue for further study to fully define the complex biology of this receptor. Of importance, modification of the fate of FPR2/ALX had significant effects on cell viability following agonist treatment. However, no obvious differences in JNK signaling were observed between FPR2/ALX and the N333-stop recycling-deficient mutant, nor in ERK 1/2 responses. This was a somewhat surprising result because one would predict a more robust JNK signal in cells exhibiting increased apoptosis. This suggests that other pro-apoptotic pathways may be involved. However, the observation of a rise in apoptotic cells when expressing the recycling-defective FPR2/ALX suggests that continued recycling is important for the homeostatic maintenance of anti-apoptotic signaling as seen by a recovery of ERK 1/2 phosphorylation in WT cells. It is noteworthy that arrestin reportedly plays a role in suppressing apoptotic signaling by a number of GPCRs including the FPR1 (42). Our data are consistent with this observation and suggest that the multiple rounds of endocytosis and recycling of the WT FPR2/ALX maintain arrestin signaling, whereas the N333-stop does not. This suggests an arrestin-mediated anti-apoptotic signal that needs further investigation, as will confirmation of the importance of receptor trafficking in more physiologically relevant preparations such as neutrophils and macrophages.

Collectively, these data suggest that, in addition to activation and cellular signaling, the endocytic trafficking of GPCRs plays an important role in how FPR2/ALX functions in the inflammatory response. It is conceivable that the design of specific “trafficking-biased” ligands that do not drive endocytosis or recycling may be more apoptotic, and hence more advantageous, in the treatment of chronic inflammation.

## Footnotes

3 The abbreviations used are:

FPR: formyl peptide receptor
GPCR: G-protein-coupled receptor
DADLE: [d-Ala-2, d-Leu-5]-enkephalin
EGFP: enhanced green fluorescence protein
ANOVA: analysis of variance
β-Arr: β-arrestin
DOR: δ-opioid receptor
β_2_AR: β_2_-adrenergic receptor.
